# Symptomatology of attention deficit, hyperactivity and defiant behavior as predictors of academic achievement

**DOI:** 10.1186/s12888-022-03714-8

**Published:** 2022-01-27

**Authors:** Jerome Flores, Alejandra Caqueo-Urízar, Verónica López, Daniel Acevedo

**Affiliations:** 1 Escuela de Psicología y Filosofía, Universidad de Tarapacá & Centro de Justicia Educacional, CJE, Avenida 18 de Septiembre 2222, Arica, Chile; 2 Instituto de Alta Investigación, Universidad de Tarapacá, Arica, Chile; 3 Escuela de Psicología, Pontificia Universidad Católica de Valparaíso & Centro de Investigación para la Educación Inclusiva, Valparaíso, Chile

**Keywords:** Attention deficit, Hyperactivity, Defiant behavior, Academic achievement, Children, Adolescents

## Abstract

**Background:**

It is essential to understand the factors that affect the academic achievement of schoolchildren, both in general and in terms of the major subsectors of each grade. Although symptoms of Attention Deficit Hyperactivity Disorder (ADHD) and Negative Defiant Disorder (NDD—which are commonly recognized as externalizing problems in childhood and adolescence—have been associated with lower academic achievement in the international literature, few studies have addressed this problem in Latin America. This study aimed to analyze the possible predictive relationship of attention problems, hyperactivity, and defiant behavior on academic achievement.

**Methods:**

We recruited a sample of 4580 schoolchildren (50.9% female, 1754 belonging to primary school, and 2826 to secondary school, ranging from 9 to 18 years old). This cross-sectional study used the scales pertaining to attention problems, hyperactivity, and challenging behavior from the Child and Adolescent Evaluation System.

**Results:**

The analysis showed that attention problems significantly affected all academic achievement areas, while hyperactivity and challenging behavior affected only some of them. The regression models explained 24% of the variability in overall academic achievement in primary school and 17% in secondary school. Other predictors included sex, age, socioeconomic level, and school attendance.

**Conclusions:**

It is important to consider this symptomatology in the design of educational interventions.

## Introduction

Although it may be debatable to consider academic achievement as the sole criterion for school success, evidence indicates that it can determine both the probability of completing formal education—and thus, the possibilities of developing one’s own life project—as well as success in higher education and even in subsequent career development [1]. Growing evidence shows that externalizing mental health problems plays a key role in academic achievement during the school years [2]. Existing literature defines externalizing mental health problems as behaviors and psychological alterations in the behavioral domain, of which the manifestations may produce conflict or harm in the environment [3]. Borba and Marin demonstrated a relationship between externalizing problems and academic achievement, showing that these problems can lead students to experience negative relationships with teachers and peers, which consequently decreases their interest and motivation to learn [4].

Attention deficit hyperactivity disorder (ADHD), and negative defiant disorder (NDD) have been recognized for decades as two types of symptom configurations of externalizing problems. ADHD, as an externalizing problem, plays an important role in the relationship between children’s mental health and academic achievement, whether considered on its own [5] or along with NDD as a comorbidity. Children and adolescents who suffer from NDD in addition to ADHD perform similarly to those with ADHD alone, but have more behavioral problems [6].

Globally, the prevalence of ADHD among children younger than 18 years old of age is estimated to be 7.2% [7]. Similarly, the prevalence of NDD varies between 3.1–3.3% [8, 9]. The situation in Chile is alarming; the last national epidemiological study in Chile established that ADHD was the most prevalent mental health problem in the infant-juvenile population with a prevalence of 15% overall, 23.9% among children aged 4–11 years, and 5.4% among adolescents aged 12–18 years. More recently, another study estimated the prevalence of ADHD to be 6.4% among 3–17-year-olds [10]. Considering these high figures, compared with international literature, the possibility of overdiagnosis of ADHD among the Chilean child and adolescent population has been questioned [11]. Such a situation would start from the school system, with principals and teachers requesting referral and diagnostic confirmation to access additional resources for their care as students with transient special educational needs under the current Chilean educational regulations [12, 13]. Meanwhile, NDD had an overall prevalence of 8.7%, broken down to 11.8 and 5.1% for each age group, respectively [14].

Existing research shows that males are more frequently diagnosed with ADHD than females. Although objective tests may not find significant differences in attention problems, parents tend to identify more attention problems among female children and teachers more disruptive behavior problems among male students [15]. NDD is also more common among males than females [16].

Previous studies find that these disorders have been associated with various negative outcomes, among both school children and adults. These outcomes include increased risk of involvement in delinquent acts, increased conflict in peer and family relationships, poorer academic achievement, dropping out of school, substance use, suicide ideation, traffic accidents, employment instability, and increased obesity [17, 18]. All of this has been associated with a substantial economic cost to society, in part due to increased utilization of various public services relative to those without ADHD [19, 20]. Another recent review of longitudinal studies included comorbidity with NDD. It finds that these children and adolescents experience problems related to psychosocial dysfunction with family, peers, and school, as well as issues of non-compliance with rules [21].

The symptoms of ADHD are continuously distributed within the population, extending ADHD-associated risk to be present among people who do not meet the diagnostic threshold [2]. It appears that when ADHD occurs in comorbidity with NDD, school psychosocial adjustment difficulties are increased among children and adolescents [6]. Females with ADHD have been found to perform better than males in language and mathematics [5]. A previous study found no differences in the areas of language and mathematics; however, it did find differences in the area of science, showing that males performed better than females [22]. Another investigation that included students with both ADHD and NDD symptomatology found no differences in academic achievement by sex [23].

Previous research considered academic achievement in different ways, mainly in terms of performance. Some used the final average score for each grade or subsector. Others used the score on standardized tests applied regularly at a national level [5]. Tests that measure academic achievement at the time of applying the test in one of the areas of interest have also been used [24]. Even parent or teacher ratings have been used as references for academic achievement [6, 25]. A combination of two of the above criteria has also been used [26].

Socioeconomic status may have a chronic disadvantageous effect on students’ academic achievement, as research consistently presents a negative relationship between socioeconomic status and academic achievement [27]. Additionally, there is evidence that professionals may experience subjective bias when dealing with minorities and vulnerable groups, diagnosing with NDD or behavioral disorder, rather than the actual ADHD presented [28].

Current evidence indicates that treatment for ADHD requires a multimodal approach [29], with cognitive-behavioral psychotherapy being consistently effective, especially in combination with pharmacotherapy [30, 31]. A decisive aspect of ensuring success is to incorporate families and educators in treatment, to thereby help them to better understand the underlying symptomatology of this disorder [30–32]. Then they can collaborate optimally with health professionals.

Nevertheless, in educational terms, the emphasis on diagnosing ADHD may be counterproductive, as students with low levels of symptomatology may experience negative consequences, such as being stigmatized in the school system, which could outweigh the potential benefit of receiving treatment [33]. Recently, a systematic review on ADHD in children and adolescents [34] found a global increase in ADHD diagnosis between 1989 and 2017; this could be related to overdiagnosis and pharmacological overtreatment associated with the medicalization of childhood [35, 36].

This systematic review of the literature identified several adverse effects associated with an ADHD diagnosis in various areas of children’s lives, including academics performance [37, 38]. In Chile, for example, a mixed-design analysis of complaints related to mistreatment and discrimination at school reported by parents and guardians to the Superintendence of Education revealed that —both nationally and in all regions of the country—more than 50% of complaints were related to ADHD diagnoses; parents referred to different experiences of what they called “educational exclusion” because of having received the diagnosis [39].

Consequently, rather than an accurate diagnosis of these disorders in students, the presence of symptomatology related to attention problems, hyperactivity, and defiant behavior may be key to understanding the factors that affect academic achievement. An existing longitudinal study found that each symptoms separately and negatively affects academic achievement among students between the ages of 7 and 16 [2]. Among ADHD symptomatology, attention problems have been found to be the most relevant to academic achievement [25, 26, 40]. Conversely, hyperactivity has not always been found to be significantly associated with academic achievement [41].

In the past, there has been some focus on specific disciplinary areas of academic achievement. A review of studies involving ADHD and mathematics academic achievement, considering specific tests —rather than grade-level academic achievement—showed a significant effect [42]. Meanwhile, in terms of of language, a longitudinal study illuminated the complex relationship between ADHD and reading achievement; high ADHD symptoms at age 5 predicted low academic achievement around the age of 9 years. However, low academic achievement at age 5 also predicted high ADHD symptoms around the age of 9 years [43].

No other studies have been found that addressed this problem outside the United States or Europe. In Latin America, a significant relationship between ADHD and academic achievement has been found within the age range of 8–17 years old, by studies in Brazil [18], Colombia [44] and Argentina [45]. In Chile, a previous study addressed the relationship between ADHD and academic achievement in language tests, using a sample of 71 students in third and fourth grade of primary school (aged 8 to 10 years); it shows a significant relationship between the factors [46]. No studies have been found that addressed the relationship between ADHD/NDD symptomatology and academic achievement in primary or secondary grades or larger sample sizes. Knowledge on this relationship in a wide age range, while considering a diversity of areas of academic achievement, would contribute to the existing knowledge and would allow the design of interventions in the school setting in a timely manner.

In the present study, we used a screening instrument that does not directly address the diagnosis of ADHD, which allows us to assess the presence of common symptomatology of this disorder, such as attention problems and hyperactivity. The same is true with respect to NDD. It should be noted that NDD is also known as Oppositional Defiant Disorder.

Consequently, the aim of this study was to analyze the possible predictive relationship between attention problems, hyperactivity, and defiant behavior on academic achievement. The research hypotheses were as follows: a) the symptomatology of these externalizing problems predicts academic achievement in different disciplinary areas, and b) attention problems are the most relevant predictors.

## Methodology

This is a cross-sectional observational study looking for correlations between ADHD/NDD symptomatology and measures of academic performance [47]..

### Sample

The sample comprised 4580 students. Participants were divided into primary and secondary school groups, and the primary and secondary samples were considered separately because different versions of the Child and Adolescent Evaluation System (*Sistema de Evaluación de Niños y Adolescentes*, SENA) instrument were applied.. The baseline sociodemographic statistics are presented in Table 1. The educational dependence of the school refers to the origin of its source of funding to operation. This can be fully provided by the state (public), partially provided by the state (subsidized) or fully provided by the parents (private).

**Table 1:** Sociodemographic baseline

	Primary	(%)	Secondary	(%)
Sex				
Boys	861	49	1443	51
Girls	893	51	1383	49
NSE				
Priority	915	52	1289	46
Preferential	416	24	793	28
Not vulnerable	423	24	744	26
DepEDU				
Public	700	40	1198	42
Subsidized	955	54	1508	53
Private	99	6	120	4
Grade				
4°	645	37		
5°	577	33		
6°	532	30		
7°			592	21
8°			580	21
9°			499	18
10°			447	16
11°			388	14
12°			320	11

NSE: Socioeconomic level of the student. DepEdu: Educational dependence of the establishment

NSE has three options: first priority (priority), second priority (preferential), and non-priority (Not vulnerable). The last one is the high NSE level

The primary school sample consisted of 1754 primary school students from public, subsidized, and private schools in Arica. A total of 49.1% were male, and 50.9% were female. The mean age was 10.1, and the standard deviation (SD) was ±0.9, with an age range of 9–12 years. It included students from grades 4–6.

The secondary school sample consisted of 2826 high school students from public, subsidized, and private schools in Arica. Of the participants, 51.1% were male, and 48.9% were female. The mean age was 14.4 (SD ±1.7), with an age range of 12–18 years. It included students from grades 7–12.

### Procedure

This study was approved by the Ethics Committee of the Universidad de Tarapacá. Next, 35 educational establishments in the city of Arica were invited to participate in the study. Informed consent was obtained from the parents after explaining the purpose and scope of the study, after which the students themselves were asked for their consent. In terms of evaluation, questionnaires were performed in groups within each grade. At least two trained interviewers answered questions, together with the teacher of the same course. The duration was approximately 45 min.

### Instruments

The SENA system, mentioned earlier, was used as an instrument in this study [48]. This instrument was developed by specialists in psychopathology and psychological assessment to help in the detection of a wide range of emotional and behavioral problems among children from 3 to 18 years old. It is noteworthy that it was developed and validated entirely in Spanish, which makes it easier for children participating in this study to understand and complete. Three scales of externalizing problems were used: attention problems, hyperactivity–impulsivity, and defiant behavior. This study used the self-reporting versions for both the primary school and secondary school groups.

Answers were provided on a Likert-type scale from 1 – “never or almost never” to 5 – “always or almost always.”The total of each dimension is the average of the dimension responses and can vary from 1 to 5. Examples are: “I find it hard to be quiet doing things” (hyperactivity), “I am easily distracted” (attention problems), “I talk back to my parents or teachers” (defiant behavior). Some items vary slightly in wording between the two versions.

The hyperactivity–impulsivity scales in both primary and secondary school questionnaires each have 10 items, while the defiant behavior scale has four items in the primary school and three in the secondary school questionnaire. In the present study, the reliability of these scales, both in Alpha and Omega, were .81 for hyperactivity-impulsivity, .83 for attention problems, and .71 for defiant behavior in primary school. Meanwhile, for secondary school, it was .80, .87 and .68 respectively. This coincides with the reliability obtained by Sánchez-Sánchez research team [49]. The SENA can be used to classify students into four zones: low risk, caution, clinical significance and extreme. Regarding the percentages in each zone, the broad results of this project are described in another study, which also includes a larger sample [50].

#### Chilean Ministry of Education SIGE database

The school management system database of the Ministry of Education collects annual data on students throughout Chile. Access to this database is restricted and students are assigned an individual code to maintain their anonymity.

The data used from this database were age, grade, sex, school attendance, school dependence, socioeconomic level, and academic achievement. Both the average school academic achievement and the averages of the main subsectors were considered to carry out a more detailed analysis. The averages per subsector considered were: 1) mathematics; 2) language; 3) natural sciences; 4) social sciences; 5) visual arts; and 6) physical education.

The socioeconomic level was classified according to the priority in the allocation of school subsidy resources granted to the schools for each student by the Ministry of Education. This has three options: first priority (priority), second priority (preferential), and non-priority Priority was 30% and refers to students belonging to the most vulnerable families who live in extreme poverty, while preferential students belong to the 80% most vulnerable [51]. The coding of the educational dependence of the school was public as 1, subsidized as 2 and private as 3.

### Statistical analysis

A descriptive analysis was performed using the study variables. Subsequently, correlation and multiple linear regression analyses were performed using SPSS version 22 sofware. Seven regression models were analyzed for each school group: primary and secondary. The only change was the dependent variable. In the first model the dependent variable is the overall performance average, while in the following six, the averages of each of the subsectors were used: language, mathematics, natural sciences, social sciences, visual arts, and physical education. The predictors considered in all regresion models were attention problems, hyperactivity–impulsivity, defiant behavior, school attendance, age, socioeconomic level, and sex (1 = male, 2 = female). Demographic variables (age, sex and socioeconomic level) and school attendance were introduced to control for their effects. This allowed us to know whether the predictors contributed in all the regression models to a similar extent.

## Results

The skewness and kurtosis of the variables were within the normal distribution range for both primary and secondary schools [52]. Table 2 presents the correlations and the means (and SDs) of the primary school group variables. It shows that the correlation of each of the externalizing problems scales was higher for general academic achievement than for each of the subsectors. The most significant association was the inverse correlation between hyperactivity and general academic achievement. Overall academic achievement was not significantly correlated with the educational dependence of the school; however, it was positively correlated with socioeconomic level, school attendance, and students’ age. None of the externalizing problems scales were significantly correlated with socioeconomic level. The inverse correlation between defiant behavior and educational dependency was found only for the latter variable. Both attention problems and hyperactivity–impulsivity were positively correlated with age. School attendance was negatively correlated with the two aforementioned symptoms..

**Table 2:** Correlations and descriptive statistics of behavioral problems and achievement in primary school

	HIP	ATE	DES	GPA	Asisten	Matem	Lengua	Cs Nat	Cs Soc	Artes V	Ed. FIS	DepEDU	Age	NSE	*M*	*SD*
HIP	–														1.9	0.7
ATE	.738^**^	–													2.3	0.8
DES	.562^**^	.493^**^	–												1.5	0.7
GPA	−.254^**^	−.359^**^	−.229^**^	–											6.1	0.5
Asisten	−.055^*^	−.093^**^	−.022	.171^**^	–										94.9	4.5
Matem	−.193^**^	−.317^**^	−.148^**^	.854^**^	.153^**^	–									5.5	0.9
Lengua	−.219^**^	−.308^**^	−.210^**^	.840^**^	.165^**^	.730^**^	–								5.6	0.8
Cs Nat	−.189^**^	−.291^**^	−.179^**^	.865^**^	.132^**^	.734^**^	.755^**^	–							5.7	0.7
Cs Soc	−.195^**^	−.298^**^	−.197^**^	.865^**^	.141^**^	.741^**^	.749^**^	.760^**^	–						5.7	0.7
Artes V	−.182^**^	−.233^**^	−.150^**^	.631^**^	.141^**^	.425^**^	.389^**^	.484^**^	.457^**^	–					6.5	0.6
Ed. FIS	−.132^**^	−.193^**^	−.136^**^	.510^**^	.085^**^	.360^**^	.356^**^	.347^**^	.405^**^	.353^**^	–				6.7	0.4
DepEDU	−.013	.018	−.049^*^	.026	−.013	.050^*^	.017	−.014	.102^**^	−.002	.152^**^	–				
Age	.041	.089^**^	.096^**^	−.259^**^	.013	−.145^**^	−.100^**^	−.228^**^	−.204^**^	−.265^**^	−.260^**^	−.033	–		10.1	0.9
NSE	−.027	−.037	−.026	.186^**^	.013	.155^**^	.218^**^	.163^**^	.214^**^	.060^*^	.125^**^	.315^**^	−.022	–		

Note: HIP: Hyperactivity. ATE: Attention problems. DES: Defiant behavior. GPA: Grade point average. Asisten: School attendance. Math: Mathematics. Lengua: Language. Cs Nat: Natural Science. Cs Soc: Social Sciences. Artes V: Visual Arts. Ed. FIS: Physical education. DepEDU: Educational School dependence. Age: Student's age. NSE: Socioeconomic level. *M* mean, *SD* standard deviation.

* *p* < 0.05. ** *p* < 0.01 (bilateral)

Table 3 presents the correlations and the means (and SDs) of each variable in the secondary school group. The sample size (*n*) used for each variable is specified, since depending on the schools’ curricula, the subsectors may vary in the number of students who took these subjects.

**Table 3:** Correlations and descriptive statistics of behavioral problems and achievement in secondary school

	HIP	ATE	DES	GPA	Asisten	Matem	Lengua	Cs Nat	Cs Soc	Artes V	Ed. FIS	DepEDU	Age	NSE	*n*	*M*	*SD*
HIP	–														2826	2.0	0.7
ATE	.699^**^	–													2826	2.4	0.8
DES	.466^**^	.432^**^	–												2826	1.6	0.7
GPA	−.122^**^	−.285^**^	−.158^**^	–											2826	5.9	0.6
Asisten	−.043^*^	−.089^**^	−.074^**^	.242^**^	–										2826	94.9	4.6
Matem	−.097^**^	−.277^**^	−.131^**^	.795^**^	.213^**^	–									2826	5.4	0.9
Lengua	−.121^**^	−.245^**^	−.103^**^	.849^**^	.195^**^	.606^**^	–								1927	5.6	0.7
Cs Nat	−.141^**^	−.278^**^	−.126^**^	.866^**^	.181^**^	.732^**^	.721^**^	–							1965	5.4	0.8
Cs Soc	−.086^**^	−.230^**^	−.120^**^	.804^**^	.177^**^	.597^**^	.709^**^	.690^**^	–						2757	5.7	0.8
Artes V	−.144^**^	−.179^**^	−.106^**^	.683^**^	.143^**^	.403^**^	.573^**^	.491^**^	.464^**^	–					1709	6.4	0.7
Ed. FIS	−.093^**^	−.123^**^	−.091^**^	.472^**^	.157^**^	.325^**^	.362^**^	.349^**^	.337^**^	.252^**^	–				1943	6.5	0.5
DepEDU	.052^**^	.055^**^	.039^*^	−.087^**^	.010	−.070^**^	−.068^**^	−.111^**^	−.136^**^	−.049^*^	.051^*^	–					
Age	.004	.030	.017	.022	−.117^**^	−.001	.019	−.110^**^	.114^**^	.212^**^	−.002	−.115^**^	–		2826	14.4	1.7
NSE	−.002	−.013	−.035	.091^**^	.019	.044^*^	.153^**^	.058^*^	.056^**^	.110^**^	.154^**^	.301^**^	.043^*^	–			

Note: HIP: Hyperactivity. ATE: Attention problems. DES: Defiant behavior. GPA: Grade point average. Asisten: School attendance. Math: Mathematics. Lengua: Language. Cs Nat: Natural Science. Cs Soc: Social Sciences. Artes V: Visual Arts. Ed. FIS: Physical education. DepEDU: Educational School dependence. Age: Student’s age. NSE: Socioeconomic level. *n* Sample size. *M* mean, *SD* standard deviation

* *p* < 0.05. ** *p* < 0.01 (bilateral)

The correlations of externalizing problems with overall academic achievement were all negative and significant, with attention problems again being the highest. However, they were lower in magnitude than in the primary school group. Academic achievement did not correlate significantly with age, but correlated positively with the educational dependence of the school, school attendance, and socioeconomic level. None of the externalizing problems correlated significantly with age or socioeconomic level. However, all of these factors correlated positively and significantly with educational dependence. School attendance correlated negatively with attention problems and hyperactivity–impulsivity, as well as with defiant behavior.

Finally, seven regression models were estimated for overall academic achievement and the main subsectors in each group. In both primary and secondary schools, academic achievement (overall or by subsector) was correlated with age, educational dependence, socioeconomic level, and school attendance, which were controlled for in all regression analyses by including them as predictors. Sex was also controlled for. Attention problems, hyperactivity–impulsivity, defiant behavior were considered as predictors of overall academic achievement and of each subsector separately (see Table 4).

**Table 4:** General and subsector regression models

Primary					Secondary				
Source^a^	Predictor	R^2^ modelo^b^	*ß*^c^ estand	*p*	Source^a^	Predictor	R^2^ modelo^b^	*ß*^c^ estand	*p*
GPA		0.237			GPA		0.171		
	HIP					HIP		.180	.000
	ATE		−.290	.000		ATE		−.368	.000
	DES		−.051	.000		DES		−.064	.001
	Asisten		.138	.000		Asisten		.218	.000
	Age		−.223	.000		Age		.063	.000
	NSE		.167	.000		NSE		.082	.000
	sexo		.075	.036		sexo		.147	.000
Matem		0.15			Matem		0.13		
	HIP		.079	.016		HIP		.195	.000
	ATE		−.348	.000		ATE		−.377	.000
	DES					DES		−.044	.027
	Asisten		.125	.000		Asisten		.183	.000
	Age		−.116	.000		Age			
	NSE		.140	.000		NSE		.035	.048
	sexo					sexo			
Lengua		.178			Lengua		.150		
	HIP		.075	.031		HIP		.129	.000
	ATE		−.286	.000		ATE		−.321	.000
	DES		−.085	.001		DES			
	Asisten		.127	.000		Asisten		.169	.000
	Age		−.062	.005		Age		.047	.028
	NSE		.204	.000		NSE		.151	.000
	sexo		.127	.000		sexo		.190	.000
Cs. Nat		.160			Cs. Nat		.127		
	HIP					HIP		.127	.000
	ATE		−.258	.000		ATE		−.355	.000
	DES					DES			
	Asisten		.108	.000		Asisten		.141	.000
	Age		−.204	.000		Age		−.092	.000
	NSE		.148	.000		NSE		.052	.014
	sexo					sexo		.108	.000
Cs. Soc		.178			Cs. Soc		.116		
	HIP		.074	.031		HIP		.170	.000
	ATE		−.281	.000		ATE		−.318	.000
	DES		−.075	.005		DES		−.049	.018
	Asisten		.117	.000		Asisten		.164	.000
	Age		−.172	.000		Age		.143	.000
	NSE		.199	.000		NSE		.046	.011
	sexo					sexo		.093	.000
Artes V		.146			Artes V		.146		
	HIP					HIP			
	ATE		−.188	.000		ATE		−.173	.000
	DES					DES			
	Asisten		.115	.000		Asisten		.154	.000
	Age		−.243	.000		Age		.217	.000
	NSE		.046	.038		NSE		.102	.000
	sexo		.129	.000		sexo		.196	.000
Ed. FIS		.112			Ed. FIS		.057		
	HIP					HIP			
	ATE		−.160	.000		ATE		−.106	.000
	DES					DES			
	Asisten		.072	.001		Asisten		.142	.000
	Age		−.244	.000		Age			
	NSE		.113	.000		NSE		.149	.000
	sexo					sexo			

Note: HIP: Hyperactivity. ATE: Attention problems. DES: Defiant behavior. GPA: Grade point average. Asisten: School attendance. Math: Mathematics. Lengua: Language. Cs. Nat: Natural Science. Cs. Soc: Social Sciences. Artes V: Visual Arts. Ed. FIS: Physical education. DepEdu: Educational School dependence. Age: Student’s age. NSE: Socioeconomic level

Note 2 A model was generated to predict both overall academic performance and each of the sub-sectors in both primary and secondary school. The predictors considered in each model are the same

Note 3 Demographic variables (age, sex, socioeconomic level, age) and school attendance were introduced to control for their effect as confounders

^a^grade point average to be predicted in each model

^b^R^2^ corrected model

^c^ß standardized Beta coefficient (ß represents the one standard deviation change in the dependent variable’s score resulting from a one standard deviation change in the independent variable)

In the primary school group, the results show that, with the exception of hyperactivity–impulsivity, the predictors explain 23.7% of the variance in overall academic achievement. Although hyperactivity–impulsivity does not contribute to explaining overall academic achievement, it is present in the prediction models of mathematics, languages, and social sciences. Attention problems are the most important predictor in almost all models, except for visual arts and physical education, where age is of greater magnitude. Defiant behavior is present in the general achievement model, as well as in language and social studies. Apart from the general achievement model, sex is only a factor in languages and visual arts, with female students obtaining higher achievements. The socioeconomic level affects all models; this shows that a higher socioeconomic level predicts better academic achievement. Age also appears in all models, with a negative predictive relationship with academic achievement. The predictive capacity of the regression models in each subsector varies from 11.2% in physical education to 17.8% for both language and social sciences.

In the secondary school group, overall academic achievement can be explained by 17.1% of all predictors. Hyperactivity–impulsivity appears in all models, except for the visual arts and physical education. Again, attention problems were the most important predictor in the first five models. Defiant behavior is present in the general achievement model, as well as in mathematics and social studies. Sex is present again in the general achievement model, where its magnitude doubles, as well as in language and visual arts. However, unlike in the primary school group, it is also present in the natural science and social science regression models. In all of them, being female predicts better academic achievement. Unlike in primary school, age appears to have a positive predictive relationship in the models, but only in the mathematics and physical education subsectors. The socioeconomic level was present in all models. The predictive capacity of the models in each subsector varied from 5.7% in physical education to 15% in languages.

The presence of collinearity between the independent variables was ruled out by the inflated variance factor, which was < 2 in all of them, which is well below the usually accepted cut-off [53].

## Discussion

The present study aimed to analyze the possible predictive relationship of attention problems, hyperactivity, and defiant behavior on academic achievement. Our results completely or partially supported the hypotheses of the study. The main finding was that the variable most closely related to academic achievement was attention problems. This was followed by hyperactivity–impulsivity, and finally, defiant behavior. However, the latter two are not always predictors. In this relationship, demographic variables (sex, age, and socioeconomic level), school attendance, and the school’s educational dependence were controlled for in the analyses.

One strength of this study is its the large sample size. Another strength was the wide age range of participants, as we considered both primary and secondary school students. Another favorable aspect was the analysis of academic performance in a broader spectrum than usual, including various subsectors of academic performance. That allows for a more complete picture of the relationship.

The analysis results partially support the first hypothesis, as each variable separately was significant in predicting some of the areas of academic achievement. Our analysis showed that attention problems is the most relevant predictor associated with overall academic achievement in some learning subsectors. This confirms the second hypothesis, and is in line with existing international literature that denotes attention problems as one of the most important externalizing mental health factors with respect to academic achievement [41]. Attention problems was the only predictor that was present in all regression models. These results are consistent with those of previous research [4, 25, 26, 44]. The conceptual explanation for why attention problems affect academic achievement requires further research; however, it is likely related to a different use of attentional resources [54] than what regular classroom teachers assume and expect among students. In contrast, hyperactivity–impulsivity was not present in all primary or secondary regression models, which is consistent with the results of previous international studies [41]. Defiant behavior was also not constant in these models, although it was constant in both primary and secondary models of overall academic achievement.

The regression model explained 6.6% more in the primary school sample than in the secondary school sample. It is possible that this is because symptomatology is more prevalent in primary school than in secondary school. It is also possible that increased academic difficulty and greater complexity in social relationships play a role in this difference [23, 55].

It is notable that different achievement areas that are not usually considered in achievement research, such as social studies, natural sciences, and especially visual arts, showed similar results to language and mathematics. The variance explained between them in the primary school sample is lower than 3%. Meanwhile, physical education remains lower, but still has predictive capacity. In the secondary school sample, the same areas also behave similarly, not varying by more than 3% among themselves. Once again, the only area that is notably different is physical education. This suggests that the academic difficulties experienced by students with ADHD/NDD symptomatology are not limited to subsectors that require greater logical-deductive resources, such as mathematics, but extend to other areas of academic achievement that require the development of sensitive competencies and fine motor skills, such as visual arts. However, the difference generated in each subsector in the predictive capacity of the models—for both primary and secondary schools—decreases by no more than 3% in mathematics, language, and natural sciences. In the social sciences, it decreases by 6.2% and in physical education by 5.5%. Meanwhile, visual arts had exactly the same value in both school groups.

It may be considered insufficient to use the final average of each subject and the general average of each grade as academic achievement indicators, rather than standardized tests that measure specific skills. However, students’ grades are those that they face in their daily context, along with their actions and reactions in their interpersonal relationships with their teachers and parents. Moreover, starting in secondary school, these grades are accumulated as part of their grade for higher education, at least in Chile.

The results suggest that, in addition to the mental health predictors in its externalizing symptomatology, there are sociodemographic factors that affect academic achievement, although not all of them in the same way and in the same directionality, referring to primary and secondary school. In line with the existing literature [27], socioeconomic level was significantly and negatively associated with academic achievement at both levels of education. Globally, this coincides with previous research that shows a positive relationship with academic achievement [43]. When analyzing its effect in terms of primary and secondary school, we find that, although it is significant in all primary and secondary models, it decreases in the latter group in terms of overall academic achievement.

In terms of students’ sex, in the overall academic achievement model, the results coincide with previous research that shows that female students experience better academic achievement than male students [15]. However, it is notable that, in the mathematical regression models of both groups, sex does not generate significant differences, which contradicts the results of a previous study that found that female students perform better in this area; we must keep in mind that, in this latter study, a mathematics-specific test was used [5]. This could indicate that there is a differential effect in terms of sex, depending on which academic achievement is being assessed. However, we must also take into account that in secondary school, a significant effect related to students’ sex appears in two models in which it did not appear in primary school (natural sciences and social sciences); moreover, its magnitude doubles in the general model. This could be explained by biological maturation as well as a cultural socialization effect [23, 55].

Age also shows complex behavior as a variable, since it appears as a significant predictor in all primary models in a negative relationship with academic achievement, indicating that, as primary students advance in their level of education, they obtain progressively worse academic grades. In contrast, in secondary school, the relationship between age and school academic achievement appears to be non-linear; in four models, it presents a positive correlation with academic achievement, but continues with a negative relationship in the natural sciences model. Mathematically, it does not appear to be a significant predictor.

It is notable that age is positively and significantly related to hyperactivity–impulsivity in the primary school group but has no relationship in the high school group. This suggests that there is a cut-off point beyond which symptoms may decline. These results coincide with those found in a previous study [56], where children diagnosed with ADHD were almost twice as high in the 10–11-year-old group than in the 8–9 year-old group. However, this study only included primary school students up to 11 years old. This is consistent with the lower prevalence of ADHD in adolescents than in children [10].

The results of this study provide clear evidence of the association between greater self-reporting of attentional difficulties and students’ own perception and lower academic achievement. In the school context, and given the negative implications associated with the (over) diagnosis of ADHD [34], the symptomatology associated with the self-perception of attentional difficulties needs to be considered on its own [2].

Despite its strengths and contributions, this study has several limitations. First, the sampling was not random, which reduces the generalizability of the results. Second, our sample was limited to a single region of northern Chile. Third, only one source of information was used; the self-reported questionnaires completed by the students themselves. However, it is necessary to consider that, from students’ self-reports, this study identified symptoms related to attention problems, hyperactivity–impulsivity, and defiant behaviors, without approaching the configuration of these symptoms as disorders or the study of their comorbidities. Finally, although there were several months’ difference between the application of the externalizing problems scales and the academic achievement results of each subsector, in general, causality in the relationship cannot be assumed.

Considering our findings, future research should include more geographical locations with randomly selected samples and more sources of information. Additionally, longitudinal studies would be desirable to understand the complex evolution of the relationships between reports of these problems from the perspective of students, as well as their teachers and parents, that challenge classroom normalization and pedagogical authority [57] and their effects on students’ academic achievement and school trajectories. This should be viewed from the perspective of their permanence, promotion, and graduation from primary and secondary education [58, 59]. However, it would be interesting to incorporate other variables that have received less attention in relation to achievement, such as the study habits of children and adolescents, as evidence has been found to differ significantly in students with and without ADHD [60].

## Conclusion

The symptomatology of externalizing problems considered in this study—attention problems, hyperactivity–impulsivity, and defiant behavior—have a significant relationship with academic achievement in general, as well as with some learning subsectors. After controlling for the effect of students’ socioeconomic level, age, sex, and school attendance, as well as hyperactivity problems and defiant behavior, self-reported attention problems were the most significant and consistent predictors of overall school academic achievement, both in primary and in secondary school. It is suggested that self-identified attention problems be addressed by the students themselves, by designing interventions within the school context that incorporate the diverse ways in which students distribute their attentional resources in the pedagogical activities planned by teachers. On the contrary, overdiagnosis of ADHD is not recommended, given the adverse effects of the consequent pharmacological overtreatment and stigmatization in the school context.

## Data Availability

The datasets used and/or analysed during the current study are available from the corresponding author on reasonable request.
